# Lysine biofortification in rice by modulating feedback inhibition of aspartate kinase and dihydrodipicolinate synthase

**DOI:** 10.1111/pbi.13478

**Published:** 2020-09-29

**Authors:** Qing‐Qing Yang, Wai‐Han Yu, Hong‐Yu Wu, Chang‐Quan Zhang, Samuel Sai‐Ming Sun, Qiao‐Quan Liu

**Affiliations:** ^1^ Key Laboratory of Crop Genomics and Molecular Breeding of Jiangsu Province/Key Laboratory of Plant Functional Genomics of the Ministry of Education College of Agriculture Yangzhou University Yangzhou China; ^2^ State Key Laboratory of Agrobiotechnology School of Life Sciences The Chinese University of Hong Kong Hong Kong China; ^3^ Key Laboratory of Crop Genetics and Physiology of Jiangsu Province/Co‐Innovation Center for Modern Production Technology of Grain Crops of Jiangsu Province/Joint International Research Laboratory of Agriculture and Agri‐Product Safety of the Ministry of Education Yangzhou University Yangzhou China

**Keywords:** rice, lysine metabolism, aspartate kinase, dihydrodipicolinate synthase, lysine feedback insensitive, biosynthetic pathway modification

## Abstract

Lysine is the main limiting essential amino acid (EAA) in the rice seeds, which is a major energy and nutrition source for humans and livestock. In higher plants, the rate‐limiting steps in lysine biosynthesis pathway are catalysed by two key enzymes, aspartate kinase (AK) and dihydrodipicolinate synthase (DHDPS), and both are extremely sensitive to feedback inhibition by lysine. In this study, two rice AK mutants (AK1 and AK2) and five DHDPS mutants (DHDPS1–DHDPS5), all single amino acid substitution, were constructed. Their protein sequences passed an allergic sequence‐based homology alignment. Mutant proteins were recombinantly expressed in *Escherichia coli*, and all were insensitive to the lysine analog S‐(2‐aminoethyl)‐l‐cysteine (AEC) at concentrations up to 12 mm. The AK and DHDPS mutants were transformed into rice, and free lysine was elevated in mature seeds of transgenic plants, especially those expressing *AK2* or *DHDPS1*, 6.6‐fold and 21.7‐fold higher than the wild‐type (WT) rice, respectively. We then engineered 35A2D1L plants by simultaneously expressing modified *AK2* and *DHDPS1*, and inhibiting rice *LKR/SDH* (lysine ketoglutaric acid reductase/saccharopine dehydropine dehydrogenase). Free lysine levels in two 35A2D1L transgenic lines were 58.5‐fold and 39.2‐fold higher than in WT and transgenic rice containing native AK and DHDPS, respectively. Total free amino acid and total protein content were also elevated in 35A2D1L transgenic rice. Additionally, agronomic performance analysis indicated that transgenic lines exhibited normal plant growth, development and seed appearance comparable to WT plants. Thus, AK and DHDPS mutants may be used to improve the nutritional quality of rice and other cereal grains.

## Introduction

Cereals play an important role in agriculture worldwide, especially rice (*Oryza sativa* L.), which contributes significantly to the global food pool and thereby helps to achieve food and nutritional security (Vasal, 2004). Lysine, the main limiting essential amino acid (EAA) in cereals, restricts the absorption and utilization of other amino acids and proteins, which leads to nutrient deficiency in humans and other animals (Cohen *et al*., 2014; Lee et al., 2001; Ufaz and Galili, 2008). Thus, enhancing the lysine content in cereal grains, especially rice, is a major interest of plant breeders aiming to improve the nutritional value of grains and prevent nutrient deficiency diseases such as kwashiorkor (Toride, 2004).

Owing to the importance of lysine, regulating the biosynthesis and catabolism of lysine is well characterized in plants (Wang *et al*., 2018). In higher plants, lysine biosynthesis involves a branch of aspartate metabolism, and two key enzymes, aspartate kinase (AK) and dihydrodipicolinate synthase (DHDPS), regulate lysine accumulation in this pathway. AK, the first limiting enzyme in this pathway, has been characterized at the molecular level in microorganisms and some higher plants (Azevedo and Lea, 2001; Matthews, 1999) including Arabidopsis, rice, barley, maize, carrot and coix (Azevedo *et al*., 1992; Frankard *et al*., 1997; Lea *et al*., 1992; Lugli *et al*., 2002; Teixeira *et al*., 1998; Wilson *et al*., 1991). However, there are at least two AK isoenzymes in plants; a lysine‐sensitive isoenzyme and a threonine‐sensitive isoenzyme (Azevedo *et al*., 1997). DHDPS catalyses the first reaction in lysine biosynthesis and is more important than AK for enhancing free lysine levels (Galili, 2002). DHDPS has also been characterized at molecular and biochemical levels in plants (Craciun *et al*., 2000; Frisch *et al*., 1991; Matthews, 1999), and it is also feedback‐inhibited by lysine or lysine analogs (Lee *et al*., 2001).

The first insight into feedback regulation of lysine biosynthesis was obtained by investigating the *Escherichia coli* (*E. coli*) gene *dapA* encoding a DHDPS enzyme that was ~20‐fold less sensitive to inhibition by lysine than a typical plant DHDPS (Lee *et al*., 2001; Shaul and Galili, 1992). An *AK* mutant gene (*lysC*) insensitive to lysine feedback was subsequently identified in bacteria (Kikuchi *et al*., 1999). A number of reports have focused on expressing *dapA* and/or mutated *AK* to enhance lysine and threonine content in plants (Galili and Amir, 2013). This approach proved successful, and more *AK* and/or *DHDPS* mutant genes are being sought in plants (Matthews, 1999). Previous studies identified natural mutants of lysine‐insensitive AK and/or DHDPS in *Corynebacterium glutamicum*, *E. coli*, barley and maize, and their sequences have single nucleotide substitutions (Matthews, 1999). In maize, two threonine‐overproducing, lysine‐insensitive AK mutants (Ask1‐LT19 and Ask2‐LT20) exhibited increased free threonine and lysine levels compared with wild‐type (WT) plants (Muehlbauer *et al*., 1994). In barley, the feedback characteristics of AK activity in mutant plants were changed so that lysine was half‐maximally inhibitory at 10 mm rather than 0.4 mm (Bright *et al*., 1982). Many attempts have been made to obtain lysine‐insensitive AK and DHDPS or lysine overproducing mutants of various plants by selecting plants resistant to the lysine analog S‐(2‐aminoethyl)‐cysteine (AEC), and some lysine overproducing mutants have been successfully generated for several species including tobacco, maize, carrot, rice and bulrush millet (Galili *et al*., 1994; Lee *et al*., 2001). However, lysine levels have only been modestly increased in these mutants. For example, rice plants were developed from resistant cells by treating with the lysine analog, and lysine content in rice seed was elevated up to 15% of total protein (Schaeffer and Sharpe, 1987; Schaeffer *et al*., 1997; Wenefrida *et al*., 2013). Unfortunately, mutants of AK and/or DHDPS that are insensitive to lysine feedback regulation have not yet been identified in rice.

The bi‐functional enzyme lysine‐ketoglutarate reductase/saccharopine dehydrogenase (LKR/SDH) was shown to play a pivotal role in the catabolism and accumulation of lysine via the lysine metabolic regulation pathway in plants (Azevedo and Lea, 2001). A series of studies have attempted to inhibit the activity of LKR/SDH, or combine overexpression of AK and/or DHDPS with interference of LKR/SDH to simultaneously enhance lysine biosynthesis and reduce lysine catabolism (Galili *et al*., 2017). In our previous studies, we expressed bacterial AK and DHDPS, and inhibited rice LKR/SDH activity, resulting in engineered and pyramid transgenic rice with ~25‐fold and ~60‐fold improvements in free lysine content in mature seeds (Long *et al*., 2013; Yang *et al*., 2016). Similar findings were also reported in plants using different strategies (Galili and Amir, 2013).

The lysine content has been successfully enhanced in cereals, Arabidopsis, tobacco, canola and soya bean (Wang *et al*., 2018). However, some unexpected effects on plant growth and/or development have been observed in lysine‐mutated and engineered plants, such as low germination rate, acute reduction in yield, low oil content, poor grain quality and dark‐brown seed phenotype (Jia *et al*., 2013; Wang *et al*., 2018; Yang *et al*., 2018, 2020). Thus, breeding crops with a high lysine content remains a major goal of plant breeders.

In the present study, in an attempt to increase lysine level, we modified the cDNAs encoding rice *AK* and *DHDPS*, and selected *AK* and *DHDPS* mutants that are less sensitive to lysine inhibition. Transgenic rice accumulated more free lysine in mature seeds harbouring mutated *AK* or *DHDPS* genes. We then identified two efficient mutants, and combined them with RNA interference (RNAi)‐mediated inhibition of LKR to construct polygenic transgenic rice that displayed a further enhancement in lysine accumulation in mature seeds.

## Results

### Selection of target sites for modification in rice AK and DHDPS enzymes

**Figure 1:**
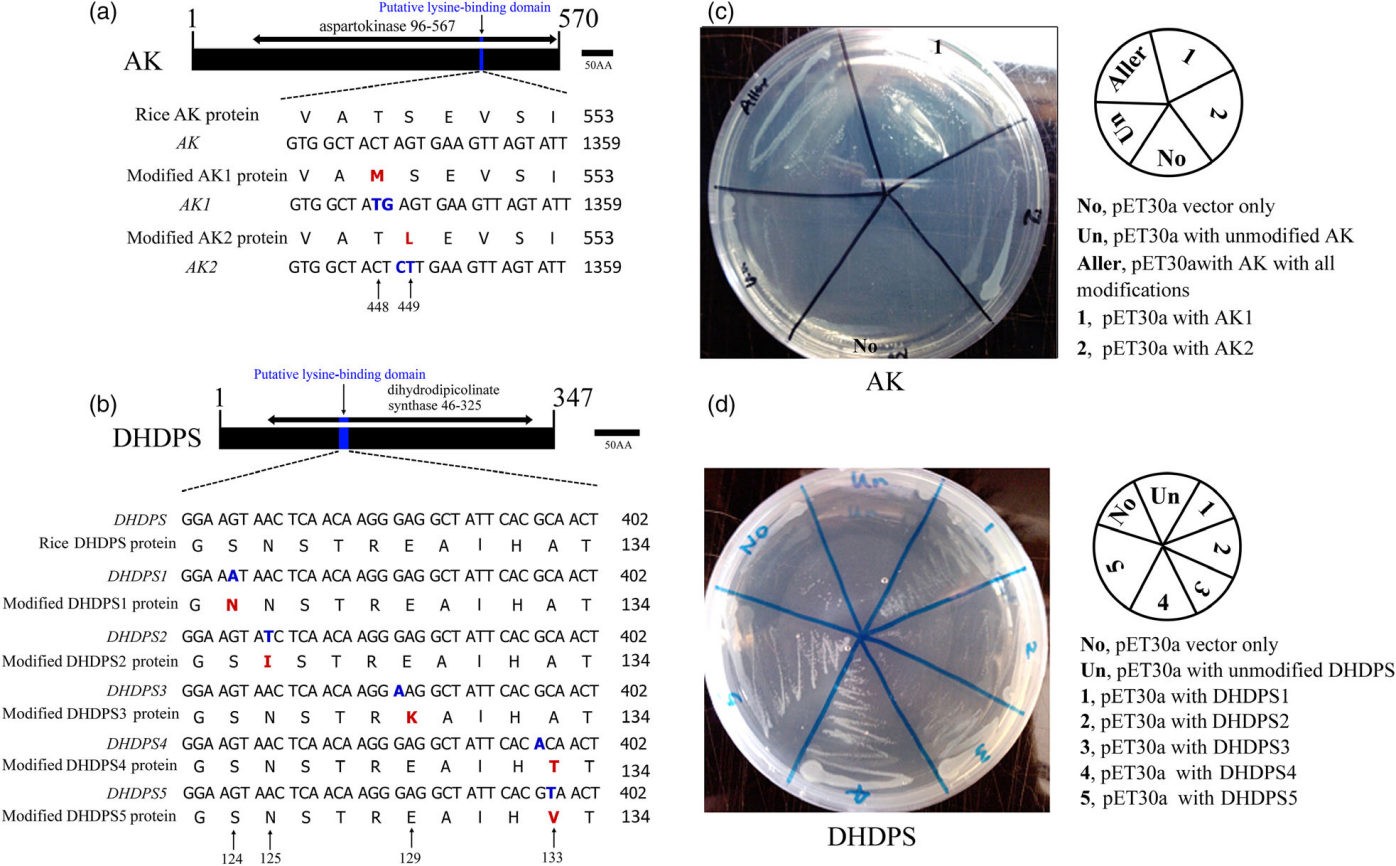
Sequence modification of rice *AK* and *DHDPS* genes, and AEC resistance testing in *E. coli* cells expressing WT and modified *AK* and *DHDPS* genes. (a) Sequence modification of rice AK. (b) Sequence modification of rice DHDPS. (c) AEC (12mM concentration) resistance testing in *E. coli* cells expressing *AK*. (d) AEC (12mM concentration) resistance testing in *E. coli* cells expressing *DHDPS*. Blue font indicates modified nucleotides, and red font indicates modified amino acids. AEC, S‐(2‐aminoethyl)‐cysteine.

Previous studies showed that feedback inhibition of the *E. coli* AKIII enzyme is a major regulatory point in lysine biosynthesis, and AKIII mutants with T344M (LysC1), S345L (LysC2) or G323D (LysC12) amino acid substitutions are insensitive to lysine feedback inhibition (Figure S1A; Kikuchi *et al*., 1999). Following comparison of the amino acid sequences of various AK enzymes in natural rice and Arabidopsis, and native and mutant *E. coli* strains (Figure S1A), we concluded that amino acid residues 448–452, corresponding to the residues 344–348 in *E. coli* AKIII, are conserved in rice AK proteins and correspond to the lysine‐binding domain (Figure S1A). Changes in this domain may affect the lysine‐insensitive AK characteristics. Therefore, Thr (T) and Ser (S) residues at positions 448 and 449 in rice AK enzyme were selected for substitution (Figure 1a).

Similarly, from the sequence alignment results of feedback‐insensitive DHDPS mutants in *E. coli*, tobacco, maize and soya bean (Matthews, 1999), it was concluded that the conserved region (residues 124–133) in rice DHDPS defines the lysine‐binding domain, and its mutation might cause the loss of lysine‐mediated inhibition of DHDPS activity (Figure S1B). Thus, five amino acids, Asn (N), Ile (I), Lys (K), Thr (T) and Val (V) at positions 124, 125, 129, 133 and 133 in the natural rice DHDPS enzyme, were selected for single amino acid substitution (Figure 1b).

### Single amino acid mutation in rice AK and DHDPS

The cDNAs encoding *AK* or *DHDPS* were cloned from rice. To obtain feedback‐insensitive AK and DHDPS by overlapping PCR, specific primers were designed for nucleotide modification (Table S1). According to the selected site, two modified *AK* clones (*AK1* and *AK2*) and five modified *DHDPS* clones (*DHDPS1*–*5*) were successfully generated via point mutations, and all mutations clustered within the predicted lysine‐binding domain (Figure 1A‐B). For AK modifications, mutation of AK1 at 448th codon (nucleotides 1343 and 1344) caused a Thr (T) to Met (M) substitution, and mutation of AK2 at 449th codon (nucleotides 1345 and 1346) resulted in a Ser (S) to Leu (L) substitution (Figure 1a). For DHDPS modifications, mutation of DHDPS1–5 resulted in G to A, A to T or C to T substitutions at nucleotides 371, 374, 385, 397 and 398, respectively, in the *DHDPS* coding region. Each nucleotide change created a single amino acid substitution: Ser (S) to Asn (N) at position 124 (DHDPS1), Asn (N) to Ile (I) at position 125 (DHDPS2), Glu (E) to Lys (K) at position 129 (DHDPS3), Ala (A) to Thr (T) at position 133 (DHDPS4) and Ala (A) to Val (V) at position 133 (DHDPS5) (Figure 1b).

In addition, the results of allergenic assessment showed that rice AK protein and its modified proteins passed the 8‐mer and 80‐mer searches, but had a small hit with a Par h I allergen (44.7% identity) in the full FASTA search (Table S2). Their E value had <3.9E‐07, and the allergen come from *Parthenium hysterophorus* and different from rice species (Table S2). The rice DHDPS and its modified proteins showed no significant alignment with any known allergen. As well as, these protein sequences identity were not likely to be cross‐reactive with other allergens. Thence, bioinformatics results predicted that rice AK and DHDPS proteins and their modified proteins had no potential allergenicity.

### AK and DHDPS mutants are resistant to feedback inhibition by lysine in *Escherichia coli*

S‐(2‐aminoethyl)‐l‐cysteine (AEC) can substitute lysine to elicit feedback inhibition of key enzymes AK and DHDPS in the aspartate metabolism pathway (Shaver *et al*., 1996; Xu *et al*., 2016). *Escherichia coli* BL21 cells expressing modified (AK1, AK2 or DHDPS1–5) or unmodified AK and DHDPS from rice were cultured on medium containing 12 mm AEC. Colonies harbouring modified *AK* or *DHDPS* genes grew normally, and better than those harbouring unmodified genes and blank controls, although the growth states differed (Figure 1c,d). Cells harbouring unmodified genes grew poorly because expression was feedback‐inhibited by the lysine analog AEC (Figure 1c,d). These results indicate reduced sensitivity of modified *AK* and *DHDPS* to lysine‐mediated feedback inhibition compared with cells expressing native rice *AK* and *DHDPS*.

### Functions of mutated AK and DHDPS in transgenic rice

**Figure 2:**
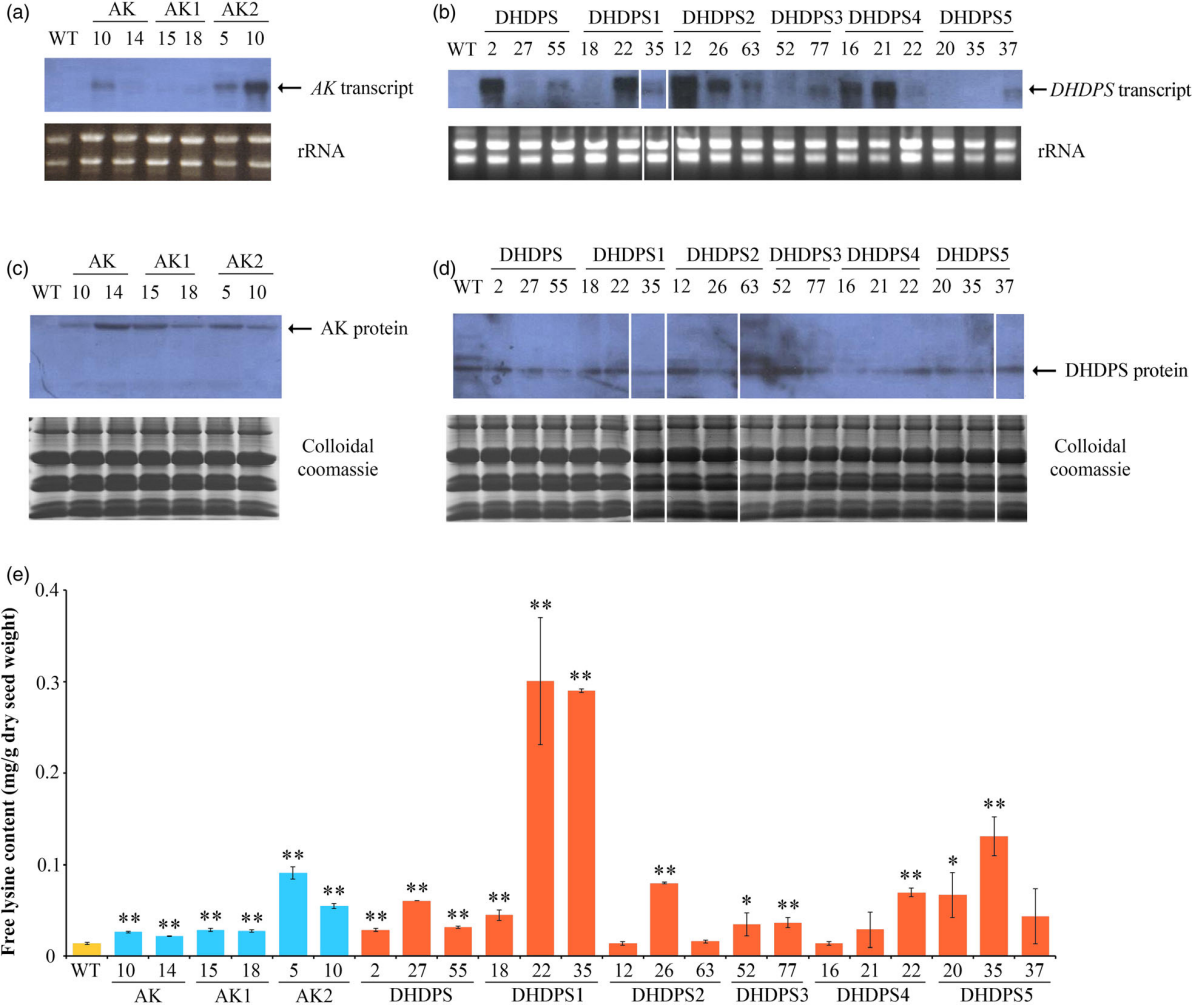
AK and DHDPS expression in developing rice seeds, and free lysine content in monofactorial transgenic and WT rice. (a, b) Northern blot analyses of rice *AK* and *DHDPS* expression in 15 DAF developing seeds. (c, d) Western blot analyses of rice AK and DHDPS expression in 15 DAF developing seeds. (e) Free lysine content (mg/g dry seed weight) in modified and unmodified *AK* and *DHDPS* transgenic and WT rice. Error bars represent SD for three biological replicates, and * and ** indicate significant difference between transgenic and WT plants with *P* < 0.05 and *P* < 0.01, respectively. The white lines represent the images from discontinuous lanes in a film or different films.

We generated nine constructs with single target gene, including two for unmodified controls with the native rice *AK* or *DHDPS* genes and seven with their modified genes (Figure S2A; Table S3), where the rice endosperm‐specific *glutelin 1* (*Gt1*) promoter were used to drive the expression of target genes in rice. These constructs were used for *Agrobacterium*‐mediated transformation to generate transgenic rice. The leaves of transgenic plants were collected for PCR analysis to confirmed target gene insertion, and more than 10 individual transgenic lines per construct were identified. Both northern and Western blotting analyses showed that rice *AK* and *DHDPS* genes were highly expressed in developing seeds at 15 days after fertilization (DAF) in transgenic rice containing modified or unmodified *AK* and *DHDPS*, and expression levels were higher than those in untransformed wild‐type (WT) rice (Figure 2a–d).

Preliminary analysis showed that the free lysine content was increased in most transgenic mature seeds, especially in transgenic lines harbouring modified AK2 and DHDPS1 (Figure 2e). The level of free lysine was significantly increased up to 6.6‐fold and 21.7‐fold in *AK2* and *DHDPS1* transgenic lines, respectively (Figure 2e). The free lysine content was also increased in AK (1.6‐ to 1.9‐fold), AK1 (2.0‐ to 2.1‐fold), DHDPS (2.0‐ to 4.4‐fold), DHDPS2 (1.0‐ to 5.8‐fold), DHDPS3 (2.5‐ to 2.6‐fold), DHDPS4 (1.0‐ to 5.0‐fold) and DHDPS5 (3.1‐ to 9.5‐fold) transgenic lines (Figure 2e). The results revealed that increasing the expression of endogenous *AK* or *DHDPS* genes can enhance free lysine levels in rice, and modification of AK2 (Ser to Leu substitution at amino acid residue 449 in rice AK) and DHDPS1 (Ser to Asn substitution at amino acid residue 124 in rice DHDPS) can significantly boost free lysine levels in mature rice seeds compared with either unmodified *AK* or *DHDPS* transgenic rice or wild‐type rice.

### Regulation of lysine metabolism by simultaneous expression of mutated AK2 and DHDPS1 in rice

Based on previous successful strategies related to lysine catabolism to manipulate free lysine accumulation in plants, we investigated whether lysine levels could be further elevated in transgenic rice by simultaneously expressing mutated AK2 and DHDPS1 (Figure S2B,C; Table S3). Constructs were generated for expressing native (35ADL) or modified (35A2D1L) rice *AK* and *DHDPS* genes, and for inhibiting expression of the *LKR/SDH* gene by *LKR*‐RNAi, all driven by the *CaMV 35S* promoter, in an attempt to simultaneously stimulate lysine biosynthesis and diminish lysine catabolism (Figure S2B,C). Over 40 independent transgenic lines were obtained using the two constructs, and transgenic lines (both 35ADL and 35A2D1L) were randomly chosen as representative lines for subsequent analysis.

**Figure 3:**
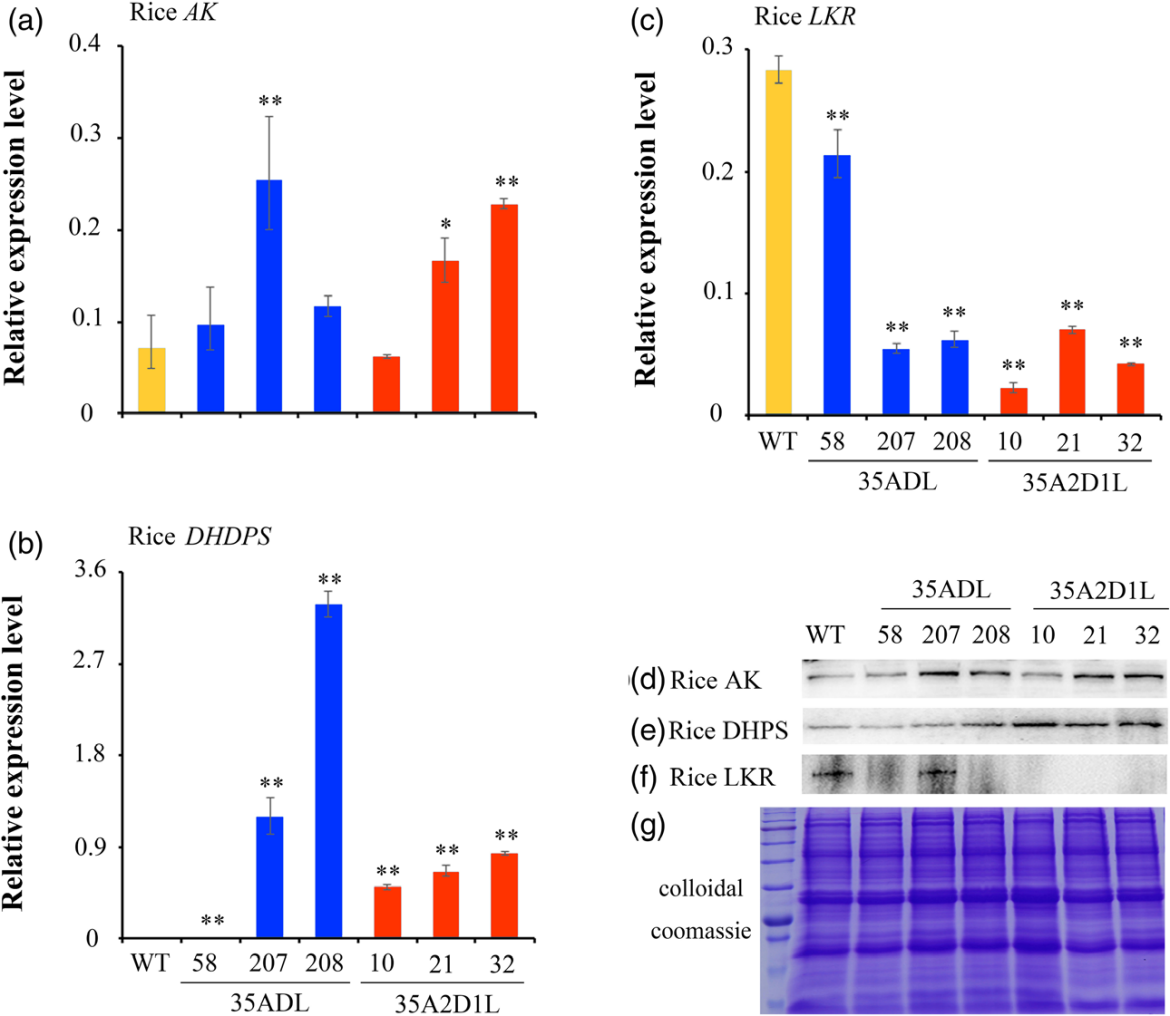
AK, DHDPS and LKR expression in developing rice seeds of polygenic transgenic and WT rice. (a–c) qRT‐PCR analyses of rice *AK*, *DHDPS* and *LKR* genes in 15 DAF developing seeds of transgenic and WT rice; (d–g) Western blot analyses of rice AK, DHDPS and LKR expression in 15 DAF developing seeds of transgenic and WT rice. Error bars represent SD for three biological replicates, and * and ** indicate significant difference between transgenic and WT plants with *P* < 0.05 and *P* < 0.01, respectively.

PCR analysis of the chosen transgenic lines demonstrated the stable integration of the transgenic cassettes in the genome of rice plants (Figure S2D–F). Rice AK and DHDPS expressions were elevated at both transcript and protein levels in 15 DAF developing seeds produced by 35ADL or 35A2D1L transgenic plants (Figure 3a–g) relative to their WT. Meanwhile, expression levels of LKR/SDH were inhibited or down‐regulated dramatically as expected in all transgenic lines according to qRT‐PCR and Western blot analyses (Figure 3a–g). These results revealed the successful expression of the introduced genes in the selected transformants.

### Enhancement of free lysine content in 35A2D1L transgenic rice

**Figure 4:**
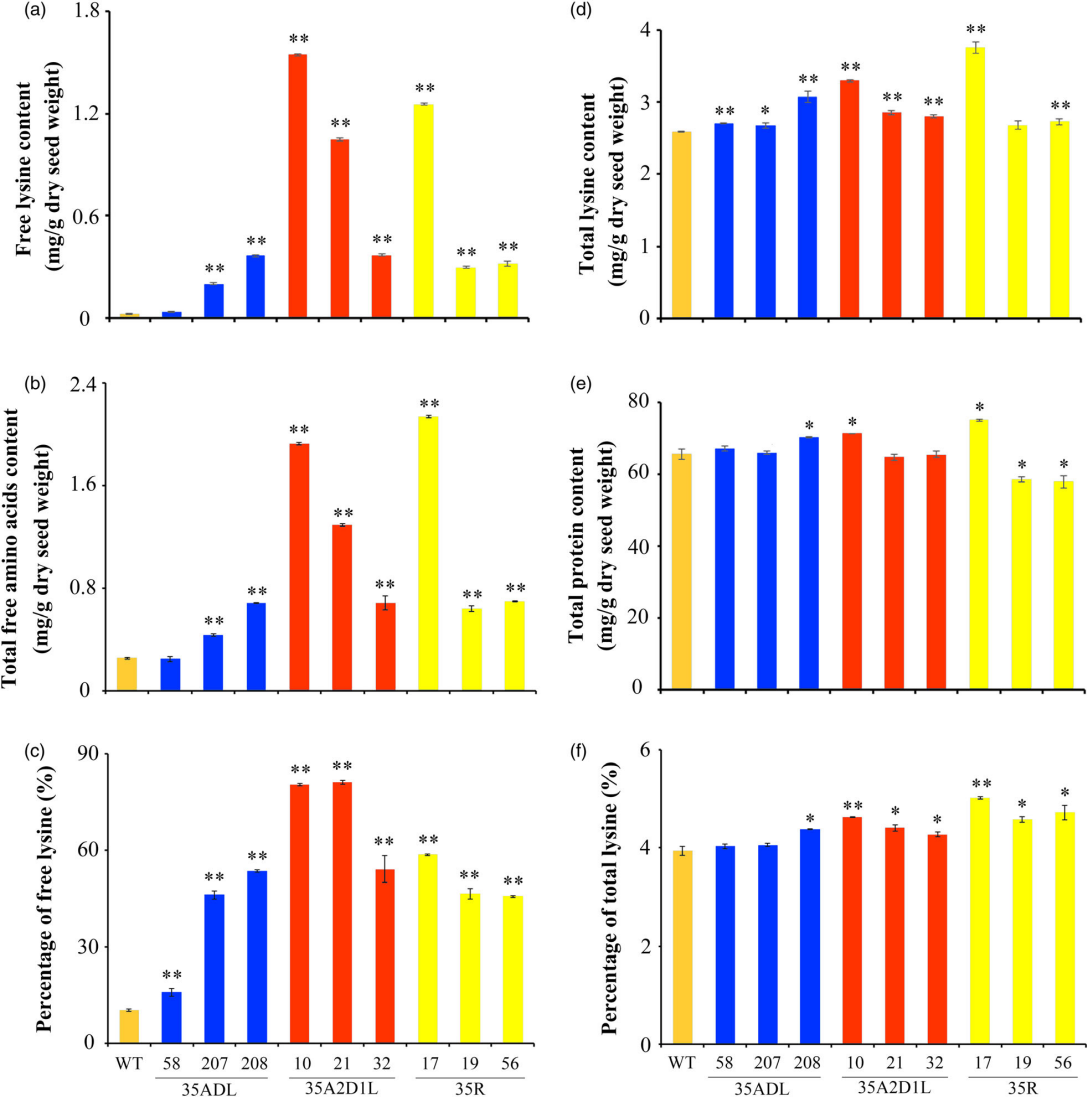
Lysine, total amino acids and protein content in mature seeds of transgenic and WT rice. (a) Free lysine content (mg/g dry seed weight). (b) Total free amino acids content (mg/g dry seed weight). (c) Proportion of free lysine among total free amino acids (weight basis). (d) Total lysine content (mg/g dry seed weight). (e) Total protein content (mg/g dry seed weight). (f) Proportion of total lysine among total proteins (weight basis). Error bars represent SD of three biological replicates, and * and ** indicate significant difference between transgenic and WT plants with *P* < 0.05 and *P* < 0.01, respectively.

To be nutritionally useful, increased lysine content in transgenic rice should be retained in seed endosperm. Thus, after natural growth and development, mature seeds were harvested and analysed, and all 35ADL transgenic lines displayed a 1.5‐ to 13.9‐fold increase in free lysine content compared with WT seeds (Figure 4a). Surprisingly, 35A2D1L transgenic lines retained significantly elevated levels of free lysine in their seeds, reaching 1544.77 ± 7.20 µg/g dry seed weight, a 58‐fold increase over the 26.41 ± 3.79 µg/g dry seed weight in WT seeds, even higher than achieved by our 35R construct generated in previous work (Figure 4a). The 35R lines are elite high lysine lines expressing bacterial *AK* and *DHDPS* genes driven by the 35S promoter (Table S3; Long *et al*., 2013). As well as, free lysine content remained higher in transgenic mature seeds at T5 generation compared to WT rice (Table S4). Additionally, the increase in free lysine content in transgenic rice seeds was associated with a significant increase in the content of all measurable free amino acids, especially in 35A2D1L transgenic lines, with levels 2.7‐ to 7.6‐fold higher than in WT seeds (Figure 4b).

The total lysine and protein contents were determined in mature seeds from WT and transgenic plants to assess whether the elevated free lysine caused changes in total lysine. As shown in Figure 4, both 35ADL and 35A2D1L transgenic lines displayed significantly higher total lysine content compared with WT rice, and it increased by 27.6% in the case of the 35A2D1L‐10 transgenic line containing the highest free lysine level (Figure 4d). The total protein content varied among different transgenic lines, and changes were correlated with the observed increase in free lysine and total lysine levels (Figure 4e). The 35A2D1L‐10 transgenic line yielded the highest total lysine and total protein (+4.62%), 17.4% higher than WT plants (Figure 4f).

Thus, expression of modified *AK2* and *DHDPS1* genes driven by the *35S* promoter combined with *LKR* interference greatly increased the free lysine content in mature seeds, and enhanced the total lysine content.

### Impact of regulating lysine metabolism on other amino acids in 35A2D1L transgenic rice

**Figure 5:**
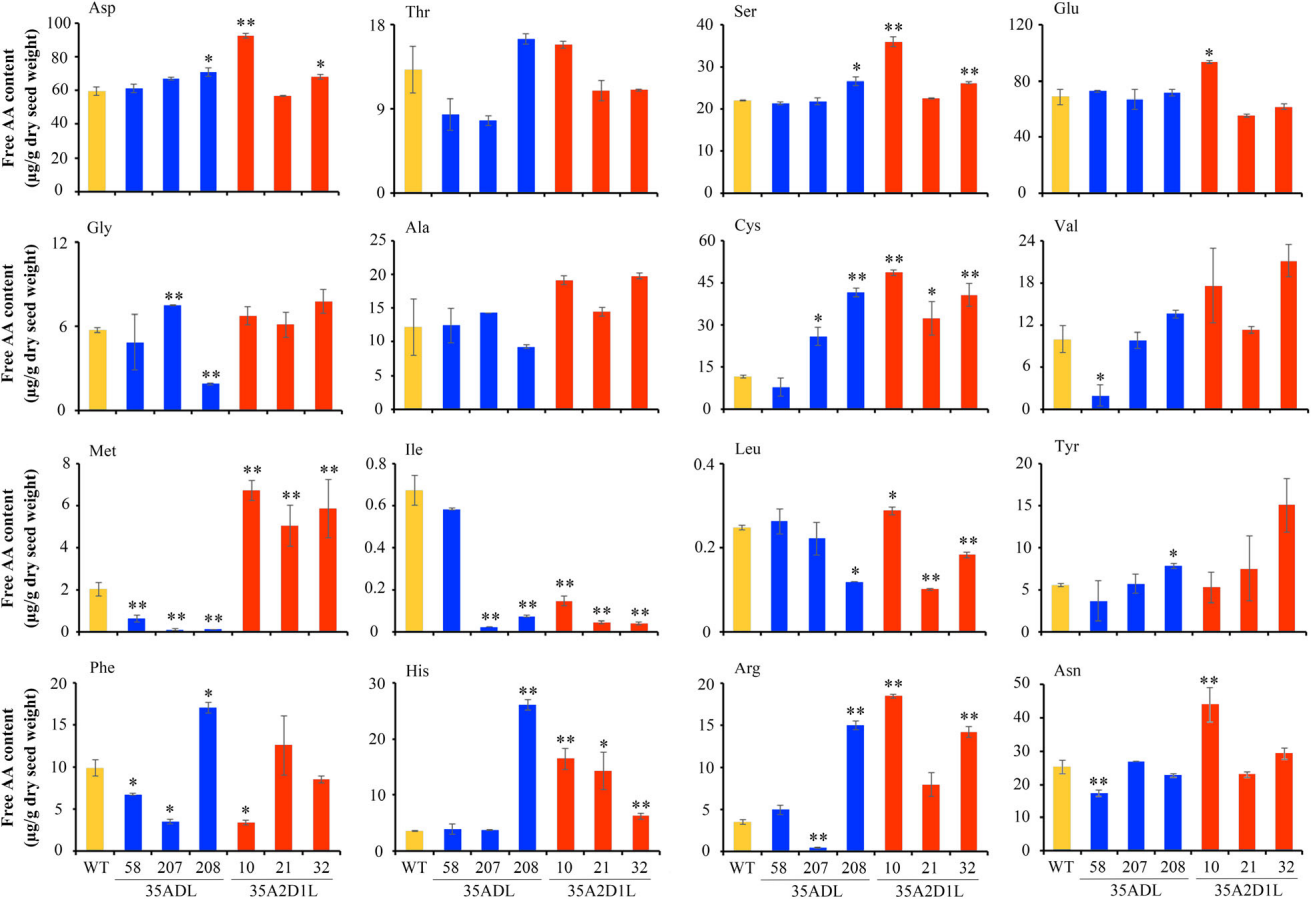
The content of other measurable free amino acids in mature seeds of transgenic and WT rice. Data are presented as μg/g dry seed weight. Error bars represent SD of three biological replicates, and * and ** indicate significant difference between transgenic and WT plants with *P* < 0.05 and *P* < 0.01, respectively.

The composition of other free amino acids (FAAs) was examined to investigate the effects of regulating lysine metabolism on their abundance in rice. Compared with WT plants, levels of most FAAs were increased, but the proportion of each amino acid among total FAAs was reduced due to the great increase in the content of total FAAs (Figure 5; Figure S3). This may be due to the increased accumulation of free lysine, since the percentage of free lysine among total FAAs was 54.1%–81.0% in 35A2D1L transgenic lines, but only 10.3% in WT plants (Figure 4c; Figure S3). These results implied that the increase in total measurable FAA levels in 35A2D1L transgenic lines is mainly due to the accumulation of free lysine in the endosperm. Asp and Glu, two major substrates of the aspartate family pathway, were elevated in 35A2D1L‐10 containing the highest free lysine content, but there was no increase in 35A2D1L‐21 and 35A2D1L‐32 transgenic lines (Figure 5). Besides, the change of free asparagine, one of the most abundant free amino acids in most cereal grains, was similar as that of Asp in 35A2D1L transgenic rice (Figure 5).

In addition, we also compared the total amounts of each measurable amino acid in seeds from 35A2D1L polygenic transgenic and WT rice (Figure S4). Surprisingly, the total content of essential amino acids, including threonine (Thr), valine (Val), methionine (Met), isoleucine (Ile), leucine (Leu), tyrosine (Tyr), phenylalanine (Phe) and histidine (His), was also increased significantly in the 35A2D1L‐10 transgenic line (Figure S4). We also calculated the amino acid score (AAS) for polygenic transgenic lines and WT plants (Table S5) based on the protein reference pattern recommended for school‐age children and adolescents (WHO, 2007). The AAS for 35A2D1L was 0.89–0.96, almost within the recommended range. These results indicated that the balance of other amino acids was surprisingly elevated in the 35A2D1L transgenic line in concert with the increase in lysine content, compared with other transgenic rice and WT controls.

### Agronomic performance of 35A2D1L transgenic rice

**Figure 6:**
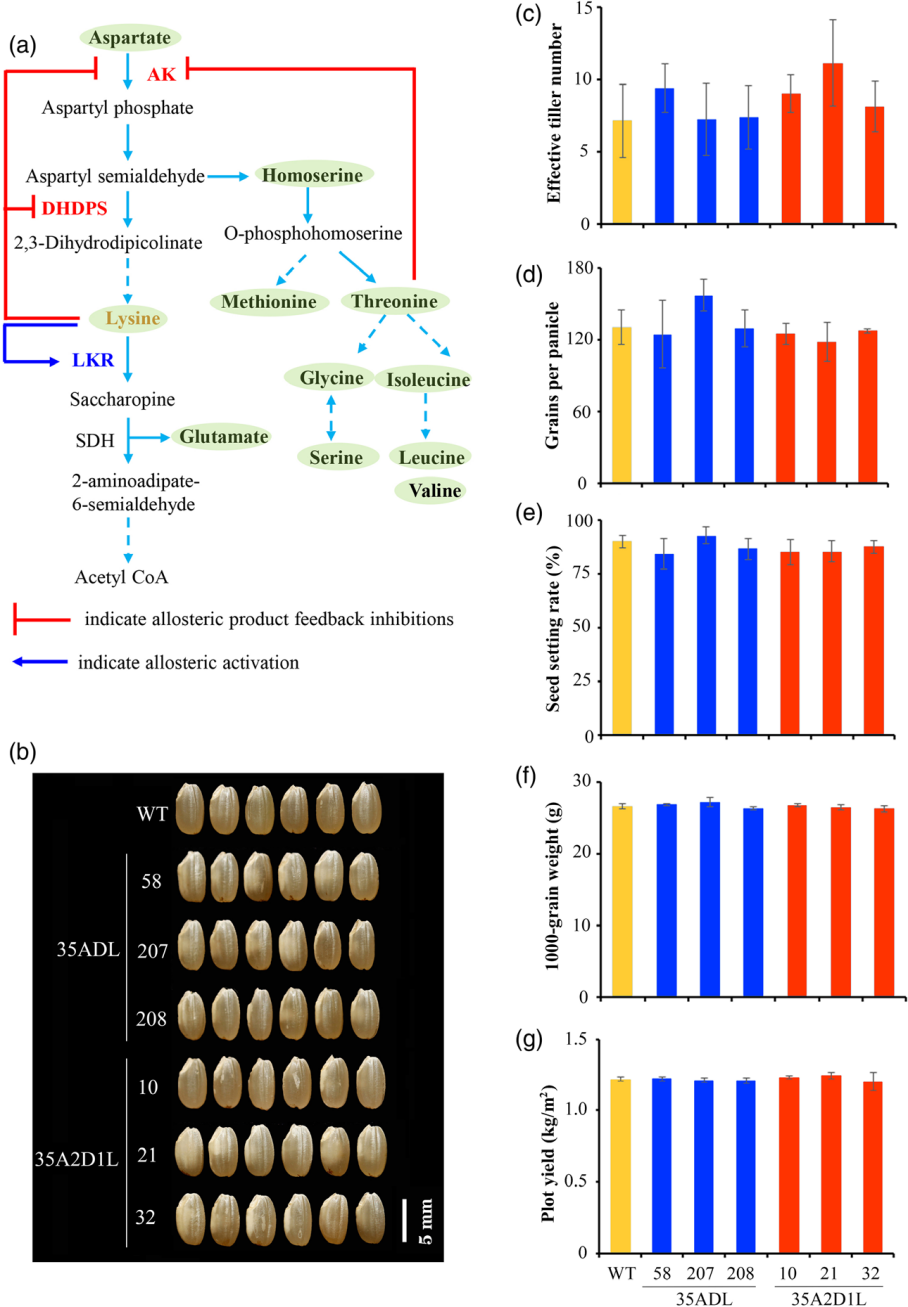
Major agronomic traits and appearance of mature seeds from transgenic and WT rice. (a) Aspartate‐derived lysine metabolism pathway in higher plants. The red lines indicate allosteric product feedback inhibitions, while blue arrow indicates allosteric activation. (b) Appearance of mature brown rice. (c) Effective tiller number, (d) Grain number per panicle. (e) Seed setting rate (%). (f) 1000‐grain weight (g). (g) Plot grain yield (kg/m^2^). Error bars represent SD of three biological replicates, and no significant difference was found between transgenic and WT plants.

Major agronomic traits were examined in the field trial, and transgenic lines exhibited normal plant growth and development for most traits as their wild type. There were no statistically significant differences in 1000‐grain weight, seed setting rate, grains per panicle, effective tiller number or plot yield between transgenic rice and WT plants (Figure 6c–g). These results indicated no obvious effect on yield upon regulating lysine metabolism by modifying rice AK and DHDPS. Interestingly, there were no obvious changes in seed chalkiness or appearance among rice lines (Figure 6b). Taken together, the above results suggested that engineering lysine metabolism in 35A2D1L transgenic lines increases the free lysine content and results in desirable trait improvements, especially in the case of the 35A2D1L‐10 transgenic line.

## Discussion

The endosperm of the maize *opaque‐2* mutant exhibited higher lysine content than normal WT kernels, providing the first insight into lysine metabolism in crops (Mertz *et al*., 1964). Subsequently, a series of mutants with high lysine content were bred or selected in *Arabidopsis*, tobacco, soya bean, rapeseed, wheat and barley (Galili and Amir, 2013). However, a high lysine rice mutant is still lacking due to the limitation of rice germplasm resources (Sun and Liu, 2004). Genetic engineering has been employed to increase the lysine content by regulating lysine metabolism, which provides an effective strategy for breeding high lysine rice (Long *et al*., 2013; Yang *et al*., 2017; Yang *et al*., 2016). Mutation of AK and DHDPS can generate lysine feedback insensitivity, but natural and engineered lysine feedback‐insensitive AK and DHDPS mutants are not yet available for rice. In the present study, we cloned rice *AK* and *DHDPS* genes and modified the corresponding enzymes to generate lysine feedback‐insensitive mutants (Figure 1). We found that the free lysine content of mature seeds was up to 21‐fold higher in transgenic lines containing modified AK and DHDPS driven by the rice *Gt1* promoter, relative to WT controls (Figure 2).

On the other hand, allergenic assessments are one stage in the process of biosafety assessments of genetically modified products. The use of bioinformatics for allergenic assessment of novel proteins in allergen databases is recommended by the FAO/WHO, the European Food Safety Authority, and the US Environmental Protection Agency (Fard *et al*., 2015). In this study, matching the 80 amino acids (domain) and 8 amino acids (epitope), in general, showed no similarity between our interest proteins (rice AK and DHDPS and their modified versions) and allergen proteins. Although AllergenOnline and Allermatch indicated that AK and its modified proteins shared 44.7% sequence identity with a Par h I allergen protein from *Parthenium hysterophorus*, the AK and its modified protein had <50% sequence identity to allergen proteins and their E value had <3.9E‐07. These results suggested no significant alignment between the AK (and its modified proteins) protein and allergen proteins in full sequence matching. Therefore, as expected, we obtained high lysine rice via the generation of lysine feedback‐insensitive mutants, which could make up for the blank of expressing endogenous lysine biosynthetic genes.

Regulation of the biosynthesis and catabolism of lysine is well characterized in plants (Galili *et al*., 2016; Wang *et al*., 2018). AK and DHDPS are key enzymes that participate in the lysine biosynthesis pathway (Galili *et al*., 2016). Various studies have aimed to enhance the lysine content in seeds by expressing AK and/or DHDPS feedback‐insensitive to lysine under constitutive or seed‐specific regulation, and marked accumulations of lysine have been achieved in Arabidopsis, barely, canola and soya bean (Wang *et al*., 2018). For example, a 2.5‐fold increase in free lysine was reported for barley seeds expressing a maize lysine feedback‐insensitive *dhps* gene under the control of the *CaMV 35S* or *Gt1* promoter, relative to WT plants (Lee *et al*., 2001). However, there was no increase in free lysine in mature seeds of 35S transgenic rice expressing bacterial lysine feedback‐insensitive *AK* and *DHDPS* genes under the control of the *CaVM 35S* promoter (Long *et al*., 2013; Yang *et al*., 2017). In the present study, endogenous rice *AK* and *DHDPS* genes were modified, resulting in two *AK* transgenic line (AK1 and AK2) and five *DHDPS* transgenic lines (DHDPS1–5) (Figure 1). The free lysine content was increased up to 21.7‐fold in mature seeds from modified *DHDPS1* transgenic lines (Figure 2), compared with a 4.0‐fold increase reported in a previous study (Lee *et al*., 2001; Long *et al*., 2013). These results indicated that single amino acid mutation of AK and DHDPS can be effective for enhancing the free lysine content, as demonstrated most effectively in AK2 and DHDPS1 transgenic lines.

Previous studies reported a 2.5‐fold increase in free lysine in seeds expressing a maize lysine feedback‐insensitive *DHDPS* gene under the control of the *CaMV 35S* or *Gt1* promoter, relative to WT plants (Lee *et al*., 2001), and similar results have been reported in other studies (Lee *et al*., 2001; Yang *et al*., 2016). This suggests that transgenic manipulation of AK and DHDPS under the control of a constitutive promoter may be more effective than using a seed‐specific promoter in terms of improving lysine biosynthesis. One explanation might be that lysine biosynthesis plays a more active role in regulating the accumulation of lysine in leaves, while lysine catabolism is the major regulatory factor in seeds (Long *et al*., 2013; Ufaz and Galili, 2008). Moreover, the *LKR/SDH* expression could be induced via the accumulation of lysine to balance amino acid flow (Stepansky *et al*., 2006; Sun and Liu, 2004; Zhu and Galili, 2003). It has been another important strategy by inhibiting or down‐regulating LKR/SDH enzyme activity for improving lysine content in plants (Hournard *et al*., 2007; Reyes *et al*., 2009). Furthermore, the expression of feedback‐insensitive *AK* and/or *DHDPS* genes combined with inhibiting the lysine catabolism pathway explosively increased free lysine content in Arabidopsis, maize and rice (Frizzi *et al*., 2008; Yang *et al*., 2016; Zhu and Galili, 2003). Therefore, simultaneous modulation of lysine biosynthesis and catabolism should be a relatively effective strategy for enhancing lysine content in plants. Indeed, in the present study, we generated the 35A2D1L transgenic line with both *CaMV 35S*‐driven expression of mutant rice *AK2* and *DHDPS1* and RNAi‐mediated inhibition of rice *LKR/SDH* expression, and the free lysine content was increased ~58‐fold in mature seeds, relative to WT plants (Figure 4), as good or even better than the excellent high lysine rice line 35R (Yang *et al*., 2016).

Great achievements have been made for high lysine maize within long‐term studies (Azevedo and Arruda, 2010). However, the *opaque‐2* mutant and Quality Protein Maize showed abnormal endosperm and starch property (Galili and Amir, 2013). A commercial high lysine maize line (LY038) was designed to express the bacterial *CordapA* gene in the embryo, and presented 19‐fold increment in free lysine content and 79% increment in total lysine content in the seeds (Lucas *et al*., 2007). Nevertheless, no phenotypic trait of the transgenic lines was reported. In our study, the free lysine level in the seeds of 35A2D1L‐10 transgenic line was 58.5‐fold and 39.2‐fold higher than that in WT or 35ADL transgenic rice with native *AK* and *DHDPS* overexpressed, respectively. Moreover, no obvious negative effect was found in these transgenic lines compared with their wild type. Therefore, these advantages will come right for the potential commercialization of such high lysine transgenic rice.

Although the free lysine content of the 35A2D1L transgenic rice seeds was greatly increased, the total lysine content only increased 27%, lower than that of the high lysine crops which expressing lysine‐rich proteins (Liu *et al*., 2015; Liu *et al*., 2016; Wong *et al*., 2015; Yu *et al*., 2005). It implied that different genetic engineering strategies may have different effects on enhancing the contents of free lysine and total lysine. Generally, there were obvious negative effects by overexpressing lysine rice proteins in plants (Liu *et al*., 2016; Wong *et al*., 2015; Yang *et al*., 2020). Thus, to avoid the obvious adverse effects, it could be widely considered to enhance the lysine content by modulating the lysine metabolism pathway in higher plants.

Due to different amino acids share the same metabolic trunk and/or acting as a synthetic substrate or intermediate, regulating metabolism of a certain amino acid may affect the accumulation of other amino acids (Figure 6a). In this study, the content of Met, the intermediate of the aspartate pathway, was significantly increased in the seeds of 35A2D1L transgenic rice relative to that of the WT (Figure 5). However, the contents of Thr and Gly, which are also derived from the aspartate pathway, were not affected in the seeds of 35A2D1L transgenic lines (Figure 5). This may be due to the overexpression of *AK2* mutant gene leading to the increase of upstream products of Met. Interestingly, the leucine and isoleucine levels were decreased in the transgenic rice seeds (Figure 5), which might be resulted from the substrate competition. Asp can be produced in plant seeds either from Glu by Asp aminotransferase or from Asn by Asn synthetase (Long *et al*., 2013). In the current study, the free Asn level was elevated in 35A2D1L‐10 transgenic rice seeds containing the highest free lysine content, but there was no increase of Asn level in 35A2D1L‐32 and 35ADL‐207 transgenic lines, and even decrease in 35A2D1L‐21, 35ADL‐58 and 35ADL‐208 transgenic lines (Figure 5). One possible reason could be that the involvement of Asp aminotransferase in the production of Asp or Asp may participate in other metabolic pathways. Additionally, the contents of essential amino acids were significantly increased or not affected in the transgenic rice (Figure S4). These results suggested that a complex regulatory network exists in modulating the overall content of these beneficial amino acids.

Notably, transgenic and mutant plants with high lysine levels exhibited some negative effects on agronomic and economic traits, characterized by retarded seed germination in *Arabidopsis* (Zhu and Galili, 2003), hardened endosperm in Quality Protein Maize (Gibbon *et al*., 2003), wrinkled seeds in soya bean (Falco *et al*., 1995), low oil content in rapeseed (Wang *et al*., 2018) and altered morphology and increased chalkiness in rice (Lee *et al*., 2001; Wong *et al*., 2015). In previous studies, we bred high free lysine transgenic rice lines HFL and 35R, and whose mature endosperm had a dark‐brown appearance (Yang *et al*., 2017; Yang *et al*., 2018). These findings indicate that the aspartate family may play multiple roles in plant metabolism, and their biological functions might differ among species. As shown in Figure 6, in this study, there was no obvious change in certain agronomic traits or the appearance of seeds, and the dark‐brown seed phenotype was not evident in the 35A2D1L transgenic rice. Previous studies showed that elevated 2‐aminoadipate from lysine catabolism may play a key role in the dark‐brown phenotype of rice endosperm (Yang *et al*., 2018). As shown in Figure S5, when the bacterial AK and DHDPS highly expressed in 35R transgenic rice, the expression levels of rice native AK and DHDPS were not different from WT plants (Long *et al*., 2013). These results suggest that the regulation of bacterial AK and DHDPS in lysine metabolism may differ from that of rice endogenous AK and DHDPS, and upstream lysine biosynthesis and downstream LKR catabolism may be relatively coordinated, resulting in insufficient 2‐aminoadipate accumulation to generate the dark‐brown phenotype. These regulatory mechanisms had been investigated using *LKR* knockout mutants and tryptophan decarboxylase (*TDC*) overexpressing transgenic lines according to our recent works on the connection between lysine metabolism and other potential pathways (Yang *et al*., 2018, 2020).

Recently, the rapid development of genome editing, including base editing via CRISPR/Cas system (Gao, 2018), has provided an important tool for precise modification of the target enzymes without foreign transgene(s). In this report, we have successfully created high lysine rice without introduction of foreign genes before the fully developed CRISPR/Cas technology. Overall, our results are promising and indicate that molecular genetic modification of the lysine synthetic pathway is a viable approach for increasing free lysine levels in important crop species such as rice.

## Experimental procedures

### Cloning and sequence modification of AK and DHDPS

Based on the cDNA sequence of rice AK (AK073189) and DHDPS (L77616) from the NCBI database, gene‐specific primers were designed (Table S1). Rice AK and DHDPS cDNAs were obtained by RT‐PCR amplification and DNA sequencing was performed to confirm construction and modification. Specific sequence modifications were performed by the overlapping PCR technique, and specific primers were designed for nucleotide modifications (Table S1).

### Allergenic assessment of candidate proteins

The allergen databases AllergenOnline (http://www.allergenonline.org/) and Allermatch (http://www.allermatch.org) were used to identify any potential sequence matches to allergen proteins. The rice original protein sequences and their modified protein sequences were searched for full FASTA, 80‐mer and 8‐mer, as suggested by the FAO/WHO guidelines for genetically modified foods (FAO and WHO, 2001). The full FASTA alignment recommendation applies to sequences of less than 80 amino acids in the FAO/WHO guidelines. And the studies showed that the efficiency of E value <3.9E‐07 in the full FASTA alignment was the same as that of sequence consistency >35% in sequence similarity ratio of 80‐mer by analysing a large number of known allergens and non‐allergens (Andre *et al*., 2009). Therefore, in this study, E < 3.9e‐07 was used as the judgment threshold for the full FASTA alignment.

The structural database of allergenic proteins (SDAP, http://fermi.utmb.edu/ SDAP/) provides rapid, cross‐referenced access to their sequences, structures and IgE epitopes that might indicate allergenic cross‐reactivity with other sequences. This alignment was performed in December 2019.

### Transgene constructs and rice transformation

Wuxiangjing 9 (WXJ9, WT), an elite *japonica* rice cultivar from China, was used for transformation in this work. *AK* and *DHDPS* genes and their mutated variants were designed and subcloned into the binary vector pSB130 (Liu, 2002), as shown in Figure S1. Nine transgene constructs were used to analyse the effects of modified AK and DHDPS in rice (Figure S1). These constructs were used for overexpression of rice AK, DHDPS and their mutated variants AK1 and AK2, and DHDPS1–5 (amino acid sequence information for mutants is shown in Figure 1), driven by the rice endosperm‐specific glutelin *Gt1* promoter.

Additionally, two transgene constructs (35ADL and 35A2D1L; Figure S1) were applied to breed high lysine rice by expressing rice *AK* (or mutated *AK2*) and *DHDPS* (or mutated *DHDPS1*) genes, and simultaneously down‐regulating the endogenous rice *LKR/SDH* gene, via three cassettes driven by the *CaMV* 35S promoter. Detailed information on the above constructs is included in Table S3. Rice calli from mature embryos were used as explants for Agrobacterium‐mediated transformation according to our previously published procedure (Liu *et al*., 1998). Stably transformed plants were regenerated after screening by PCR (Figure S2) with transgene‐specific primers (Table S1). All selected transgenic lines were homozygous for the transgenes and were grown in a greenhouse at the Chinese University of Hong Kong or in paddy fields at Yangzhou University (Yangzhou, Jiangsu Province, China).

### AEC resistance in Escherichia coli‐expressing modified AK and DHDPS

AEC (S‐(2‐aminoethyl)‐l‐cysteine), a toxic lysine analog structurally similar to lysine, can compete with lysine for incorporation into proteins, and only mutants overproducing lysine or with defective AEC uptake can survive in the presence of this compound (Galili, 1995). Herein, 12 mm AEC was added to the growing medium of *E. coli* BL21 cells expressing modified or unmodified AK and DHDPS. These constructs were cloned by digesting the pET‐30a vector (Novagen, Darmstadt, Germany) with restriction enzymes *Bam* HI and *Sac* I, then ligating the PCR‐amplified and digested modified and unmodified *AK* or *DHDPS* gene. PCR amplification was performed using an ABI PRISM dRhodamine Terminator Cycle Sequencing Kit (Applied Biosystems, Foster City, CA, USA), and analysed using an ABI PRISM 3100 Genetic Analyzer (Applied Biosystems, ABI) as described in the user manual.

### Analysis of RNA and protein expressions

Total RNAs were isolated from developing seeds at 15 days after flowering (DAF) using TRIzolRNA (Invitrogen, Carlsbad, CA) and purified by treatment with DNase I. First‐strand cDNA was generated using Perfect Real Time PrimeScript RT reagent (TaKaRa, Japan). The rice *actin* gene was used as an internal control to measure the relative expression levels of rice *AK* and *DHDPS*, bacterial *AK* and *DHDPS*, and rice *LKR/SDH* genes. Gene‐specific primers used for qRT‐PCR are listed in Table S1. The absolute values (M values) of the slope difference between the reference gene and the target genes were calculated for *AK*, *DHDPS* and *LKR*, respectively. Because the M value (0.040, 0.045 and 0.058, respectively) calculated based on these experiments was less than 0.1, the data were analysed according to the ΔΔcycle threshold (*C*_t_) method (Livak and Schmittgen, 2001) using actin as the CT normalizer. Δ*C*_t_ = *C*_t_(target) − *C*_t_(actin), while ΔΔ*C*_t_ = Δ*C*_t_(test sample) − Δ*C*_t_(control sample). Relative gene expression was analysed by the ΔΔ*C*_t_ method.

For northern blot analysis, developing total RNA was separated on a 1% agarose/formaldehyde gel and transferred to a nylon membrane, and hybridization and detection were performed as described previously (Long *et al*., 2013). Total seed proteins were extracted from developing seeds as previously described (Long *et al*., 2013). Proteins were detected by Western blotting using antibodies specific for rice AK, DHDPS and LKR, or bacterial AK and DHDPS.

### Grain component analyses

Mature seeds were shelled, milled and processed into flour for grain component analysis (Zhu *et al*., 2010). The contents of free and total amino acids, both based on the dry rice seeds, were determined as previously described (Yang *et al*., 2016). Briefly, the free amino acids were extracted from rice flours by ultrasound assisted, and the total amino acids were isolated by hydrolysed rice flours with 6 N HCl. These treated samples were then dissolved in Na‐S™ buffer and finally filtered through a 0.45‐μm nylon membrane syringe filter (Pall Life Sciences, Port Washington, NY, USA) for injection and analysis by HPLC (high‐performance liquid chromatography) using an L8900 amino acid analyser (Hitachi, Tokyo, Japan). The measurement of seed total protein contents was performed according to a previous report (Zhou *et al*., 2020). Crude protein content was estimated by Kjeldahl method via a nitrogen determiner (Foss Tecator Kjeltec 2300; Tecator AB, Hoganas, Sweden), and the nitrogen content was converted to protein content by multiplying the former by 5.95. Three biological replicates were designed for each sample.

### Field trials and agronomic trait investigation

Transgenic and non‐transgenic WT plants were grown at in the experimental field in Yangzhou University (Yangzhou, Jiangsu Province, China) with permission for small‐scale field trials under genetically modified safety supervision during the summer of years 2013–2019. A three randomized block design was adopted, and each plot contained six rows, with 10 plants per row and a spacing of 15 cm between plants and 15 cm between rows. Rice plants were grown under the same climate and management conditions. Major agronomic traits and plot yield were investigated after maturity, and mature seeds were harvested for grain component analysis.

### Statistical analysis

Results are presented as the mean ± standard deviation (SD). Comparison of multiple transgenic and WT plants was performed by *t*‐test analysis using SPSS 17.0 for windows (SPSS Inc., Chicago, IL, USA). Asterisks * and ** indicate statistical significance between transgenic and WT plants at *P* < 0.05 and *P* < 0.01, respectively.

## Conflict of interest

The authors declare no conflicts of interest.

## Author contributions

Q.Y., W.Y., H.W. and C.Z. and performed the experiments; Q.Y, S.S.S. and Q.L. designed the experiments; Q.Y., W.Y., S.S.S. and Q.L. analysed the data; Q.Y. and Q.L. wrote the paper. All authors have commented on the manuscript and approved the final manuscript.

## Supporting information


**Figure S1** Sequence Alignments of the putative lysine‐binding domain from native AK and DHDPS and their mutants.
**Figure S2** Transgene constructs for expressing modified or unmodified AK and DHDPS in rice and PCR analyses of transgenic plants.
**Figure S3** Comparison of the proportion (by weight) of other individual free amino acids among total measurable free amino acids in mature seeds of transgenic and WT rice.
**Figure S4** The contents of total essential amino acids in mature seeds of transgenic and WT rice.
**Figure S5** The expression of AK, DHDPS and LKR in developing rice seeds of transgenic and WT plants.
**Table S1** Primers used in this study.
**Table S2** Sequence identity after full searching of candidate proteins in allergen databases of AllergenOnline and Allermatch (E value < 1).
**Table S3** The information of chimeric genes for production of transgenic rice used in this study.
**Table S4** Free lysine content in mature seeds of transgenic and WT rice.
**Table S5** Proposed scores for essential amino acids in transgenic and WT rice.
