# Supplementary material for: Lysine biofortification in rice by modulating feedback inhibition of aspartate kinase and dihydrodipicolinate synthase
Source: Plant Biotechnol J. 2020 Sep 29;19(3):490–501. doi: 10.1111/pbi.13478 (PMC7955878; doi:10.1111/pbi.13478)
Supplement: Supplementary file 1 — Figure S1 Sequence Alignments of the putative lysine‐binding domain from native AK and DHDPS and their mutants. Figure S2 Transgene constructs for expressing modified or unmodified AK and DHDPS in rice and PCR analyses of transgenic plants. Figure S3 Comparison of the proportion (by weight) of other individual free amino acids among total measurable free amino acids in mature seeds of transgenic and WT rice. Figure S4 The contents of total essential amino acids in mature seeds of transgenic and WT rice. Figure S5 The expression of AK, DHDPS and LKR in developing rice seeds of transgenic and WT plants. Table S1 Primers used in this study. Table S2 Sequence identity after full searching of candidate proteins in allergen databases of AllergenOnline and Allermatch (E value < 1). Table S3 The information of chimeric genes for production of transgenic rice used in this study. Table S4 Free lysine content in mature seeds of transgenic and WT rice. Table S5 Proposed scores for essential amino acids in transgenic and WT rice. [file PBI-19-490-s001.docx]

**Supplementary Figures**

**
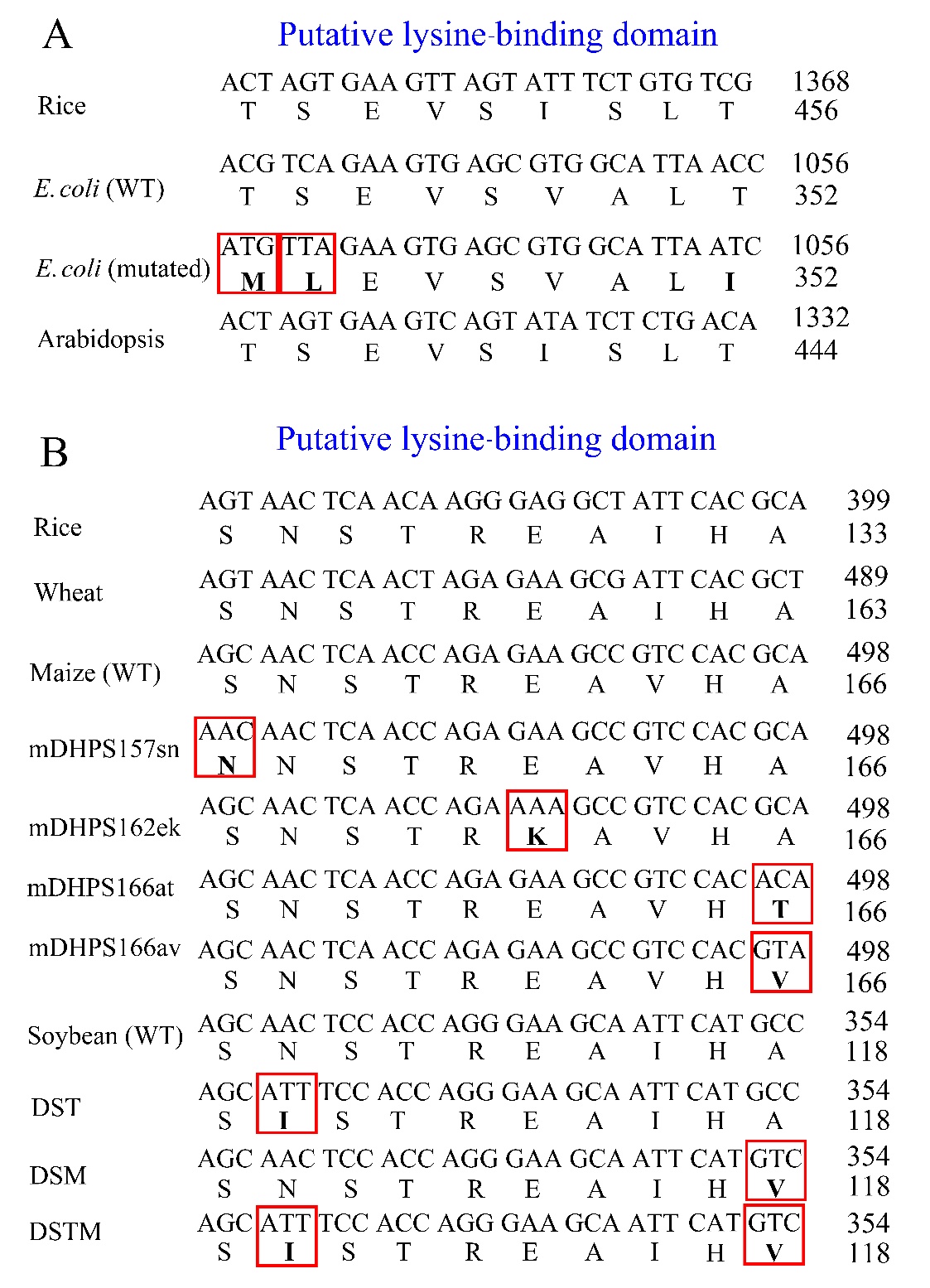
**

**Supplementary Figure S1.** Sequence Alignments of the putative lysine-binding domain from native AK and DHDPS and their mutants. (A) Alignment of the AK partial sequences from rice, Arabidopsis and *E. coli*. (B) Alignment of the DHDPS partial sequences from rice, wheat, maize, soybean. mDHPS157sn, mDHPS162ek, mDHPS166at and mDHPS166av represent four DHDPS mutants of maize, while DST, DSM and DSTM are the mutants of soybean. Red boxes indicate mutated codons. The locations of the aligned sequences were indicated on the right.


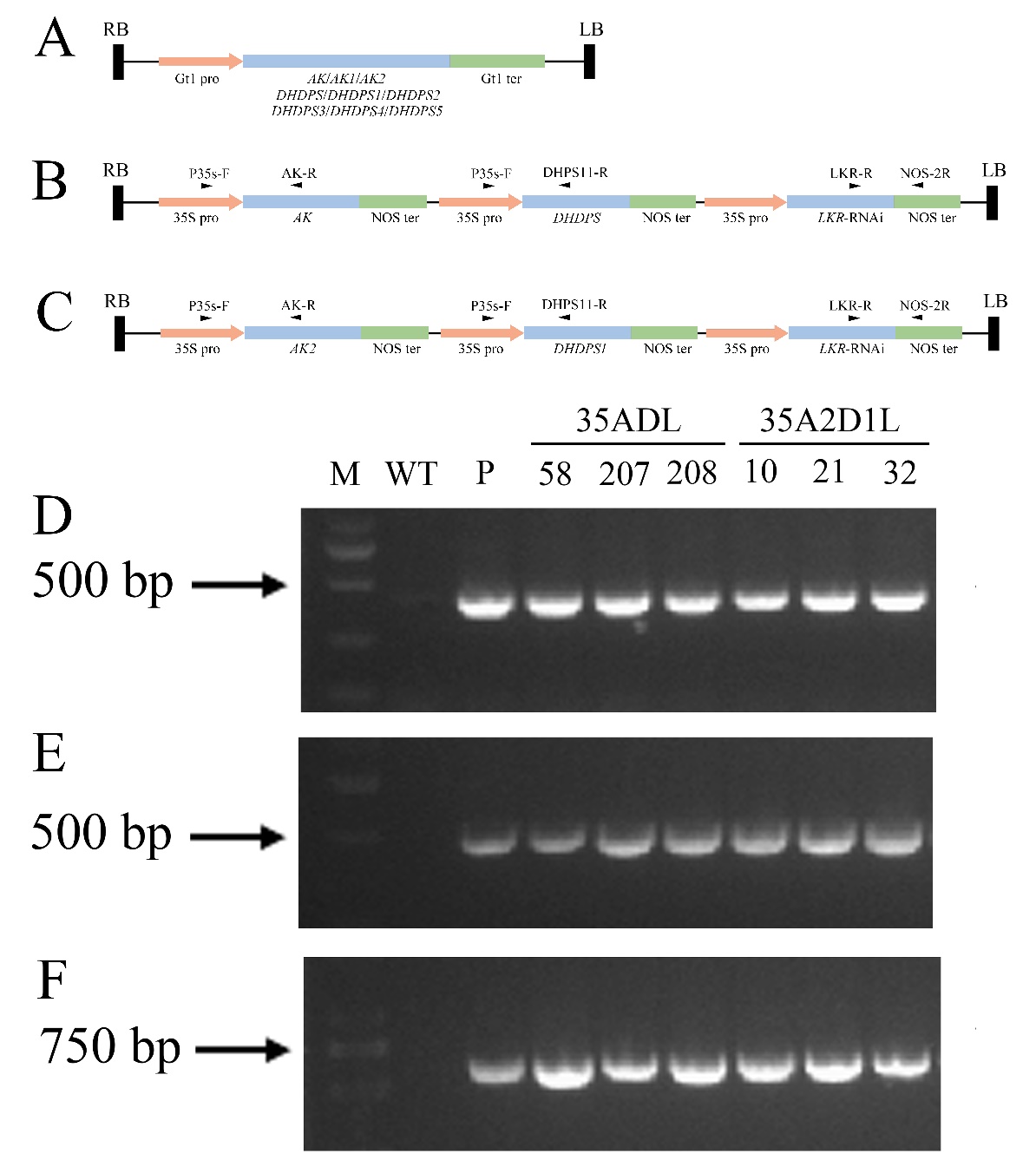


**Supplementary Figure S2.** Transgene constructs for expressing modified or unmodified AK and DHDPS in rice and PCR analyses of transgenic plants. (A–C) Only one T-DNA region of each constructs were shown for expressing single modified or unmodified AK and DHDPS. (A) Simultaneous expressing native AK and DHDPS and RNAi-inhibiting LKR/SDH (35ADL). (B) Simultaneous expressing modified AK2 and DHDPS1 and RNAi-inhibiting LKR/SDH (35A2D1L). (C) RB and LB are the right and left borders of the T-DNA, respectively. *Gt1 pro* and *Gt1 ter* represent the promoter and terminator of rice glutelin (*Gt1*) gene, respectively. *35S pro* means the *CaMV 35S* promoter. *LKR*-RNAi means the RNA interference structure for rice *LKR/SDH* gene. (D–F) PCR analyses of the *AK* (P35s-F/AK-R), *DHDPS* (P35s-F/DHPS11-R) and *LKR/SDH* (LKR-R/NOS-2R) transgenes in transgenic plants (35ADL and 35A2D1L). The locations of PCR primers were indicated in panels B and C. M, standard molecular markers; WT, wild type rice; P, plasmids as positive controls. 35ADL and 35A2D1L, transgenic rice lines derived from the constructs B and C, respectively.


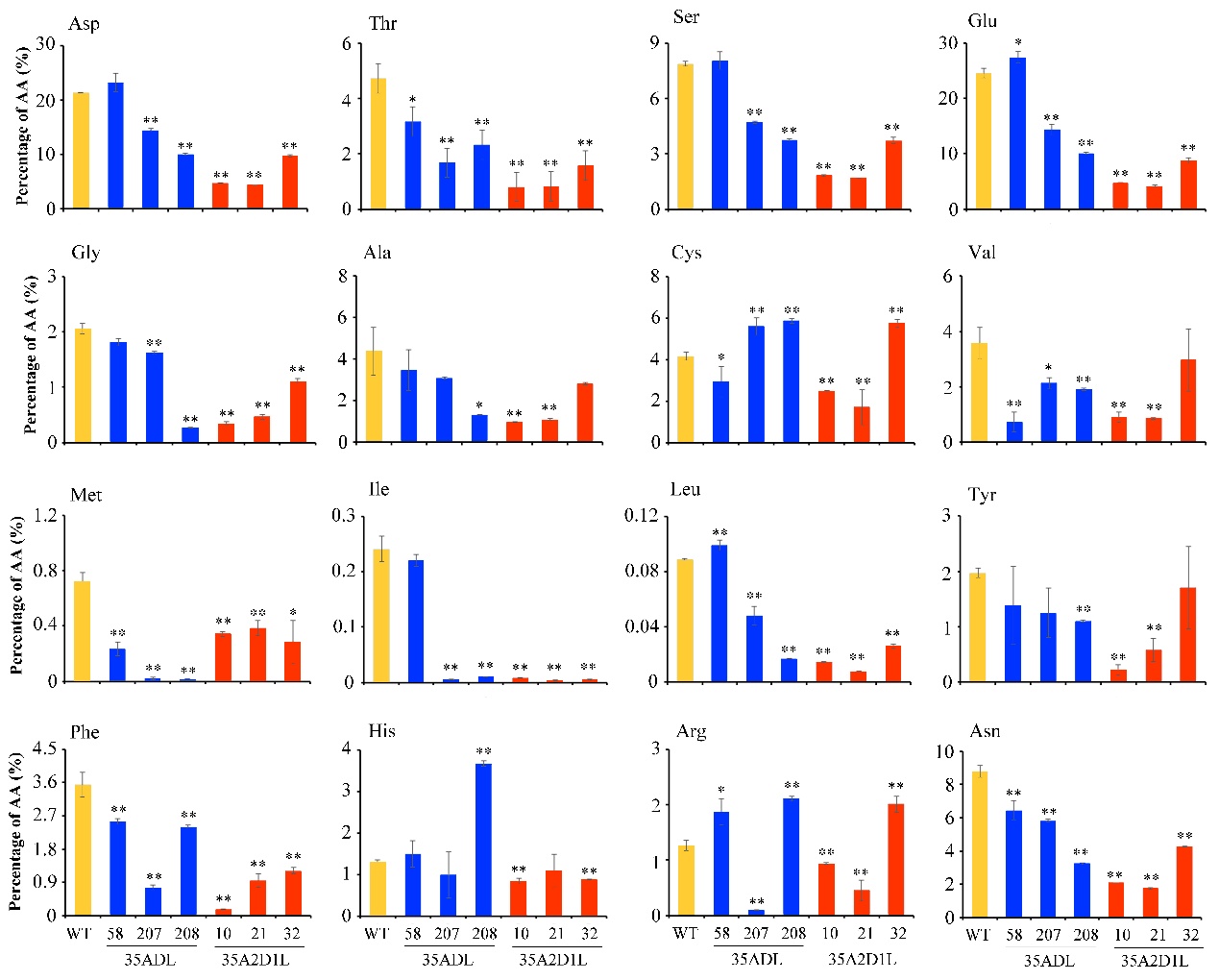


**Supplementary Figure S3.** Comparison of the proportion (by weight) of other individual free amino acids among total measurable free amino acids in mature seeds of transgenic and WT rice. Error bars represent SD for three biological replicates, * and ** indicate significant difference between transgenic and WT plants with *P* < 0.05 and *P* < 0.01, respectively.

**
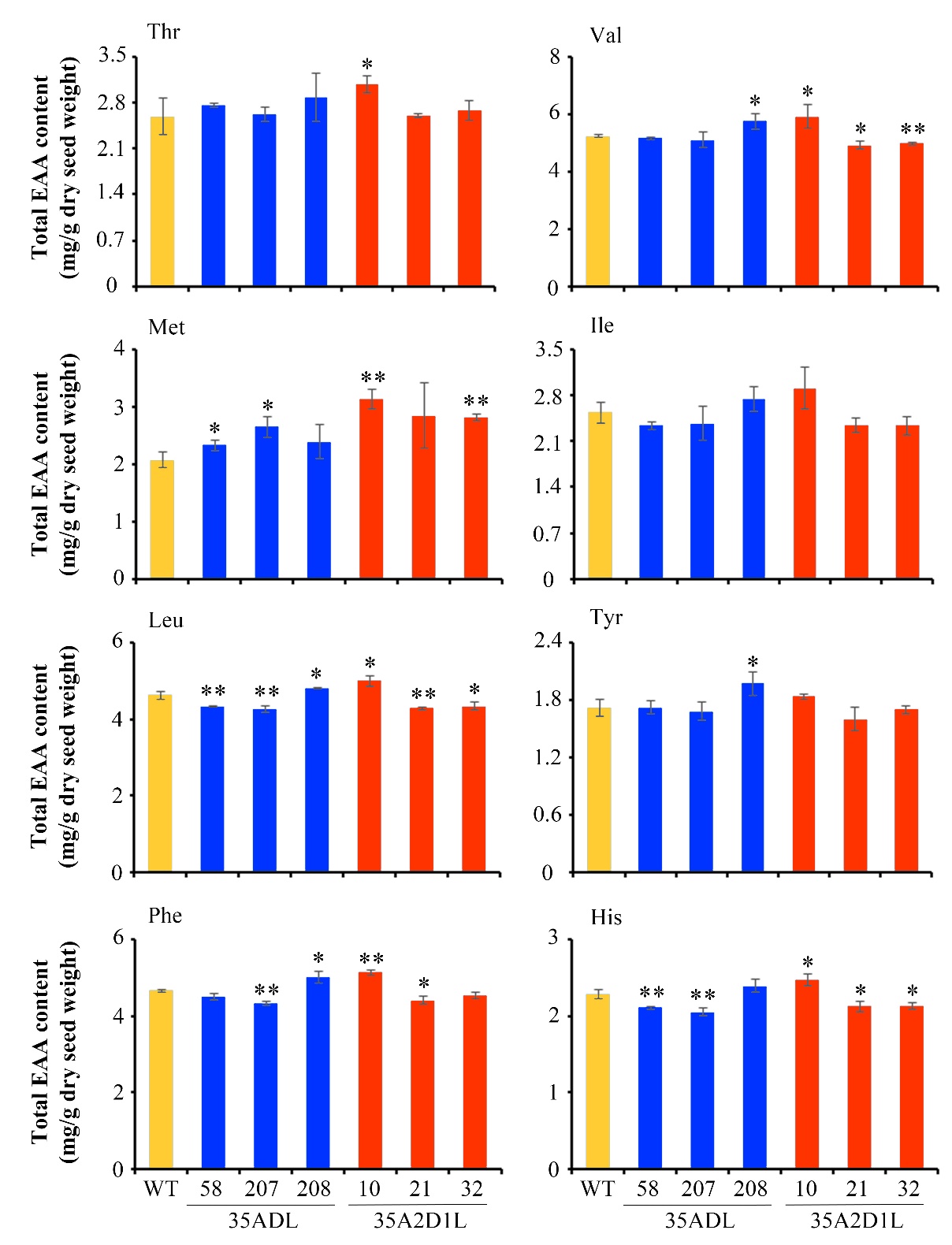
**

**Supplementary Figure S4.** The contents of total essential amino acids in mature seeds of transgenic and WT rice. Data are presented as μg/g dry seed weight. Error bars represent SD of three biological replicates, * and ** indicate significant difference between transgenic and WT plants with *P* < 0.05 and *P* < 0.01, respectively.


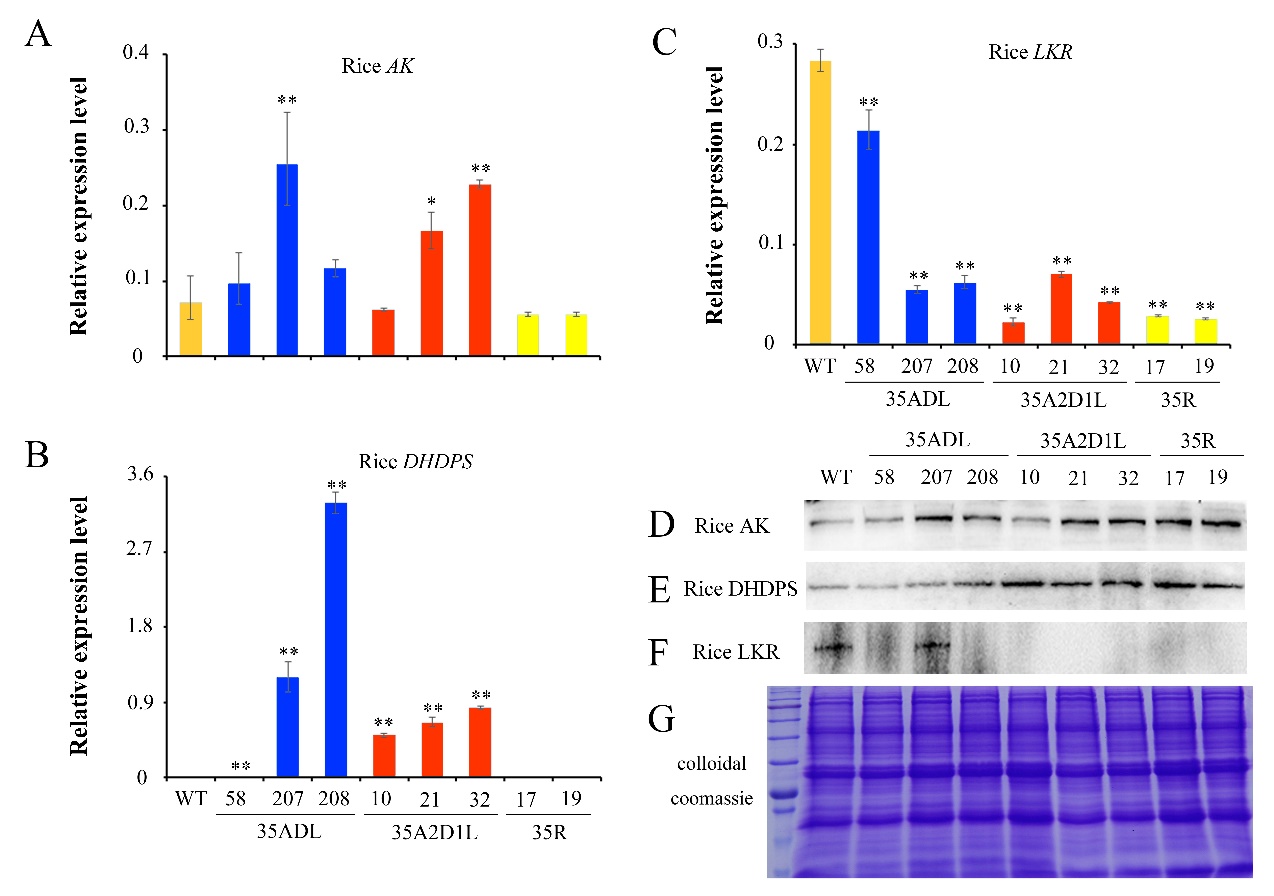


**Supplementary Figure S5.** The expression of AK, DHDPS and LKR in developing rice seeds of transgenic and WT plants. (A−C) qRT-PCR analyses of rice *AK*, *DHDPS* and *LKR* genes in developing seeds (15 DAF). (D−F) Western blot analyses of rice AK, DHDPS and LKR expression in developing seeds (15DAF). (G) The colloidal coomassie staining of total seed proteins after SDS-PAGE. 35R-17 and -19 are two transgenic rice lines with bacterial *AK* and *DHDPS* genes and *LKR*-RNAi construct (Table S3) (Yang et al., 2016). Error bars represent SD for three replicates, * and ** indicate significant difference between transgenic and WT plants with *P* < 0.05 and *P* < 0.01, respectively.

**Supplementary Tables**

**Supplementary Table S1.** Primers used in this study.

| **Primer name** | **Nucleotide sequence (5'→3')** |
| --- | --- |
| AK-Forward | AGGATCCATGGCGATCGCTCTCCGATT |
| AK-Reverse | AGAGCTCTCAGCTCATGTGAAGGGCTT |
| DHPS-Forward | AGGATCCATGCAACACTCTGACACAG |
| DHPS-Reverse | AGAGCTCTTAATACCTACTGATCAAC |
| AK1-Forward | CTGTAGATTGTGTGGCTATGAAGTATTTC |
| AK1-Reverse | GAAATACTAACTTCACTCATAGCCACAATCTACAG |
| AK2-Forward | TAGATTGTGTGGCTACTCTTGAAGTTAGTATTTCTGT |
| AK2-Reverse | ACAGAAATACTAACTTCAAGAGTAGCCACACAATCTA |
| DHPS1-Forward | GGCAACACAGGAAATAACTCAACAAGG |
| DHPS1-Reverse | CCTTGTTGAGTTATTTCCTGTGTTGCC |
| DHPS2-Forward | ACACAGGAAGTATCTCAACAAGGGAG |
| DHPS2-Reverse | CTCCCTTGTTGAGATACTTCCTGTGT |
| DHPS3-Forward | TAACTCAACAAGGAAGGCTATTCACGCA |
| DHPS3-Reverse | TGCGTGAATAGCCTTCCTTGTTGAGTTA |
| DHPS4-Forward | GAGGCTATTCACACAACTGAGCAGGG |
| DHPS4-Reverse | CCCTGCTCAGTTGTGTGAATAGCCTC |
| DHPS5-Forward | GAGGCTATTCACGTAACTGAGCAGGG |
| DHPS5-Reverse | CCCTGCTCAGTTACGTGAATAGCCTC |
| LKR-R | CCTTAGCTGAGGCCAATCTAG |
| NOS-2R | ATTGCGGGACTCTAATCATAAAAAC |
| DHPS11-R | CGTAGTAAGGATTGATGTGG |
| AK-F | CCATCTACTTCAGCAGAGGGCA |
| AK-R | CGAACTTCATCACCACGCTCA |
| GT1-2R | GGAACGGGAACACAAAAATAGAAAG |
| GT1 | GCATTCAGTTCATTAGTCCT |
| P35s-F | TCCTTCGCAAGACCCTTCCTC |
| DHPS-2 | TTACAGCAAACCGGCATG |
| Akrt-F | CTGACACTTCGGACCACTGACTACC |
| Akrt-R | ACACCATCAACATCTTTCCATACCTG |
| DHPSrt-R | CCCTGCTCAGTTGCGTGAATAG |
| DHPSrt-F | GTAGTGAAGTGAAAAATCGGACATC |
| LKRrt-F | ACTCTTCAATGCTTGTAACATCTCC |
| LKRrt-R | AGTAGGGTTGCTTGGTGCTTT |
| EDHPS-RT1 | TTCTTGCGACGGACGATTGTAGTAA |
| EDHPS-RT2 | GATGAAAAAGGTAATGTCTGTCGGG |
| AK1-RT1 | AGGAAGCACAGGTTATGGCTGGATG |
| AK1-RT2 | AGTGAGCGTGGCATTAACCCTTGAT |

**Supplementary Table S2.** Sequence identity after full searching of candidate proteins in allergen databases of AllergenOnline and Allermatch (E value < 1)

| **Databases** | **Protein** | **Allergen** | **Organism** | **Length (aa)** | **Maximum Identity (%)** | **E value** | **Algorithm** |
| --- | --- | --- | --- | --- | --- | --- | --- |
| **AllergenOnline** | AK | Putative Par h I precursor | Parthenium hysterophorus | 570 | 44.7 | 0.8 | FASTA 36.3.8g |
|  | AK1 | Putative Par h I precursor | Parthenium hysterophorus | 570 | 44.7 | 0.8 | FASTA 36.3.8g |
|  | AK2 | Putative Par h I precursor | Parthenium hysterophorus | 570 | 44.7 | 0.8 | FASTA 36.3.8g |
|  | DHDPS | No | No | 347 | No | No | FASTA 36.3.8g |
|  | DHDPS1 | No | No | 347 | No | No | FASTA 36.3.8g |
|  | DHDPS2 | No | No | 347 | No | No | FASTA 36.3.8g |
|  | DHDPS3 | No | No | 347 | No | No | FASTA 36.3.8g |
|  | DHDPS4 | No | No | 347 | No | No | FASTA 36.3.8g |
|  | DHDPS5 | No | No | 347 | No | No | FASTA 36.3.8g |
| **Allermatch** | AK | Par h 1.0101 | Parthenium hysterophorus | 570 | 44.7 | 0.76 | FASTA 3.8 |
|  | AK1 | Par h 1.0101 | Parthenium hysterophorus | 570 | 44.7 | 0.76 | FASTA 3.8 |
|  | AK2 | Par h 1.0101 | Parthenium hysterophorus | 570 | 44.7 | 0.76 | FASTA 3.8 |
|  | DHDPS | No | No | 347 | No | No | FASTA 3.8 |
|  | DHDPS1 | No | No | 347 | No | No | FASTA 3.8 |
|  | DHDPS2 | No | No | 347 | No | No | FASTA 3.8 |
|  | DHDPS3 | No | No | 347 | No | No | FASTA 3.8 |
|  | DHDPS4 | No | No | 347 | No | No | FASTA 3.8 |
|  | DHDPS5 | No | No | 347 | No | No | FASTA 3.8 |

**Supplementary Table S3.** The information of chimeric genes for production of transgenic rice used in this study.

| **Transgenic lines** | **Constructs** | **Note** |
| --- | --- | --- |
| AK | Gt1::AK | Rice endogenous AK and DHDPS genes |
| AK1 | Gt1::AK1 |  |
| AK2 | Gt1::AK2 |  |
| DHDPS | Gt1::DHDPS |  |
| DHDPS1 | Gt1::DHDPS1 |  |
| DHDPS2 | Gt1::DHDPS2 |  |
| DHDPS3 | Gt1::DHDPS3 |  |
| DHDPS4 | Gt1::DHDPS4 |  |
| DHDPS5 | Gt1::DHDPS5 |  |
| 35A2D1 | 35S::AK2+35S::DHDPS1+35S::LKR-RNAi |  |
| 35ADL | 35S::AK+35S::DHDPS+35S::LKR-RNAi |  |
| 35R | 35S::Bacterial AK+35S::Bacterial DHDPS+Gt1::LKR-RNAi | Bacterial AK and DHDPS genes |

**Supplementary Table S4**. Free lysine content in mature grains of transgenic and WT rice.

| **Transgenic line/WT** | **Free lysine content (μg/g dry seed weight)** |
| --- | --- |
| WT | 16.53±3.09 |
| 35ADL-58 | 51.37±3.67 * |
| 35ADL-207 | 262.18±7.85 * |
| 35ADL-208 | 478.50±7.63 * |
| 35A2D1L-10 | 1281.49±35.43 * |
| 35A2D1L-21 | 894.91±37.99 * |
| 35A2D1L-32 | 293.14±10.46 * |

The asterisks (*) indicate statistical significance between transgenic and WT plants at *p* <0.05.

**Supplementary Table S5.** Proposed scores for essential amino acids in transgenic and WT rice.

| **Amino acid** | **WT** | **35ADL** | | |  | **35A2D1L** | | | **Reference (g/kg protein)#** |
| --- | --- | --- | --- | --- | --- | --- | --- | --- | --- |
|  |  | **58** | **207** | **208** |  | **10** | **21** | **32** |  |
| Lysine | 0.82 | 0.84 | 0.85 | 0.91 |  | 0.96 | 0.92 | 0.89 | 48 |
| Histidine | 2.18 | 1.97 | 1.95 | 2.13 |  | 2.16 | 2.05 | 2.04 | 16 |
| Isoleucine | 1.29 | 1.16 | 1.2 | 1.3 |  | 1.36 | 1.21 | 1.19 | 30 |
| Leucine | 1.16 | 1.06 | 1.06 | 1.12 |  | 1.15 | 1.09 | 1.09 | 61 |
| Phenylalanine + tyrosine | 2.37 | 2.27 | 2.22 | 2.42 |  | 2.39 | 2.26 | 2.32 | 41 |
| Threonine | 1.58 | 1.65 | 1.6 | 1.64 |  | 1.73 | 1.61 | 1.64 | 25 |
| Valine | 2 | 1.93 | 1.94 | 2.05 |  | 2.08 | 1.9 | 1.9 | 40 |

# The proposed amino acid scoring pattern was based on the WHO 2007 report for school-aged children and adolescents.
